# Serum beta2-microglobulin and peripheral blood eosinophils for the assessment of severity and prognosis with omicron variant COVID-19 infection

**DOI:** 10.3389/fmolb.2024.1476080

**Published:** 2024-11-20

**Authors:** Jie Tan, Hanxi Fang, Xiao Hu, Ming Yue, Junling Yang

**Affiliations:** Department of Respiratory Medicine, The Second Hospital of Jilin University, Changchun, China

**Keywords:** COVID-19, omicron, β2-MG, eosinophil, prognosis

## Abstract

**Background:**

The Omicron variant’s high transmissibility has made it the most widespread novel coronavirus variant. Elevated serum β2-MG levels from viral infections and EOS’ role in viral clearance have garnered attention. However, their predictive value for Omicron’s severity and prognosis needs further exploration.

**Methods:**

This retrospective study included 424 patients with confirmed COVID-19 Omicron variant admitted to the Second Hospital of Jilin University in Changchun, China, of whom 128 experienced in-hospital mortality. Patients were divided into high and low groups according to β2-MG and EOS levels; the relationship between disease severity and patient prognosis was analyzed.

**Results:**

Our findings showed that severe-to-critical Omicron patients had higher β2-MG levels than mild-normal patients. Conversely, EOS levels were higher in mild-moderate cases. Both β2-MG and EOS levels normalized when Omicron patients tested negative for nucleic acid. Deceased Omicron patients had significantly lower pre-mortem EOS levels. Elevated β2-MG and lower EOS levels correlated with reduced overall survival. Multivariate COX regression analysis indicated that elevated β2-MG was an independent adverse prognostic factor for Omicron patients.

**Conclusion:**

High serum β2-MG levels and low eosinophil levels upon admission correlate with omicron variant severity and prognosis. β2-MG is an independent risk factor for poor outcomes in omicron patients.

## Introduction

The coronavirus disease 2019 (COVID-19), caused by SARS-CoV-2, has had a profound global impact (Wiersinga et al., 2020). Following multiple mutations, the Omicron variant, currently the most concerning variant (VOC), demonstrates notably higher transmissibility than previous SARS-CoV-2 strains (Fosbøl et al., 2020). Omicron infections can trigger systemic inflammatory responses, abnormal coagulation dysfunction, multiple organ damage, and other pathophysiologic changes, leading to a poor prognosis for patients with its severe or critical form. In late 2022 and early 2023, Changchun, China, experienced an omicron BA.5 and BF.7 epidemic. Public health organizations and medical institutions have effectively prevented and managed COVID-19, while systematic and organized treatment has been conducted for patients with omicron infection. Understanding COVID-19’s progression remains crucial for diagnosing and addressing emerging coronaviral diseases globally.

β2-Microglobulin (β2-MG) is a non-glycosylated small molecule protein, serving as the light chain for major histocompatibility complex class I antigens (Berggård and Bearn, 1968). It is widely found in plasma, urine, cerebrospinal fluid, and other body fluids (Ma et al., 2012). β2-MG is produced by all nucleated cells, with epithelial cells, mesenchymal cells, and lymphocytes being primary sources. Under normal conditions, serum β2-MG remains consistently low. β2-MG has been investigated as a new indicator of inflammation in ischemic-hypoxic encephalopathy, lower respiratory tract infections, and other diseases (Cai et al., 2020; Carreras et al., 2023). Elevated serum β2-microglobulin (β2-MG) levels are associated with viral infections, including human immunodeficiency virus, EBV, CMV, and influenza (Cooper et al., 1984; Zipeto et al., 2018).

Eosinophils (EOS), a type of blood leukocyte commonly assessed in routine blood count tests (Klion et al., 2020), play a role in identifying and predicting outcomes in infectious diseases (Karakonstantis et al., 2019). They aid in diagnosing allergic diseases (Eng and DeFelice, 2016), often rising during bronchial asthma episodes (Wardlaw et al., 2000). Research indicates that patients with comorbid bronchial asthma are less likely to be infected with COVID-19, which may be related to viral resistance in asthma and allergic diseases (Liu et al., 2020). Eosinophilia on admission may help in early diagnosis of COVID-19 infection (Soni, 2021). In addition, low eosinophil levels, although not significantly correlated with patient admission to the intensive care unit (ICU), can predict death in ICU patients (Xuan et al., 2022).

In our study, elevated serum β2-MG levels and decreased EOS count in peripheral blood correlated with symptom grading and poor prognosis in omicron patients. We aimed to predict patient outcomes using a combined β2-MG and eosinophil rating.

## Materials and methods

### Subjects

**FIGURE 1:**
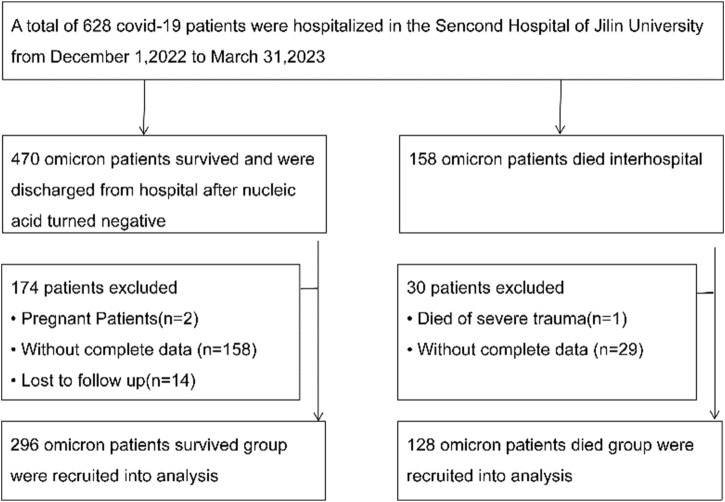
Screening of patients with Omicron variant COVID-19.

We reviewed electronic medical records of omicron patients hospitalized between 1 December 2022, to 31 March 2023, at the Second Hospital of Jilin University. The “Diagnosis and Treatment Protocol for COVID-19 Patients (tentative 8th edition)” (Diagnosis and Treatment Protocol for, 2022) outlines the criteria for classifying hospitalized patients as mild-to-moderate or severe. A total of 424 hospitalized patients, diagnosed with COVID-19 via laboratory tests, were included in this study (Figure 1). Viral nucleic acid testing confirmed SARS-CoV-2 infection in every patient, each testing positive for SARS-CoV-2 RNA. Based on the valid sequencing sequences of the new coronavirus genes released, it was determined that the patients were all infected with the Omicron variant. Of these, 247 were male and 177 were female, with a median age is 72 (62.25–80.00) years. All participants underwent a 120-day follow-up, with death defined as the adverse outcome. The main study outcome was in-hospital mortality. As of 31 March 2023, there were 128 deaths and 296 discharges. Fresh blood specimens were collected within 24 h of admission and sent to the laboratory for testing.

The Ethics Committee of the Second Hospital of Jilin University reviewed the study in line with the principles outlined in the Declaration of Helsinki, approving the retrospective review of medical records (No. 2023215). Given the study’s minimal risk to the participants, to the committee waived the requirement for individual informed consent.

### Serum β2-MG determination

Blood samples were collected from the peripheral veins of subjects who had strictly fasted for more than 8 h. Serum β2-MG levels were determined using a latex-enhanced immunoturbidimetric assay. These were performed according to the instructions of Zybio’s β2-MG kit and using an automated biochemical analyzer (Labospect 008 AS).

### Complete blood count tests

Peripheral blood samples collected from all subjects were anticoagulated using EDTA. Peripheral blood cells were counted to obtain absolute counts using a Sysmex XN fully automated hematology analyzer (Sysmex Corporation, Japan).

### Statistical analysis

SPSS 26.0 and GraphPad Prism 9.5.0 were used for statistical analysis. Categorical variables were presented as frequencies and percentages, while continuous variables were expressed as mean ± standard deviation or interquartile range. Differences between continuous variables across groups were assessed using the Student’s t-test or Mann–Whitney *U* test. The χ2 test was used to compare differences in count data between groups. The log-rank test and Kaplan-Meier analysis were used to compare overall survival (OS). Cox proportional risk regression was used for univariate and multivariate analyses. All the above analyses were done with SPSS 26.0. Correlation analysis between the two sets of data was done by applying the application GraphPad Prism 9.5.0. The Forestplot package of R software (Version 4.3.1) was applied to draw forest plots. Statistical significance was set at *p* < 0.05.

## Results

### Patient characteristics

A total of 424 patients with omicron variant COVID-19 were followed up for 120 days, with 128 deaths. The median overall survival (OS) duration was 98.07 days (2–120 days). Among them, 76 exhibited mild to moderate symptoms, while 348 had severe symptoms based on clinical guidelines. The mild-to-moderate group included 41 males and 35 females [median age: 70.50 years (56.75–82 years)]. The severe group had 206 males and 142 females [median age: 73 years (64–80 years)]. Upon evaluation at discharge, patients were segmented into a survival cohort of 296 patients and a deceased group comprising 128. The survival group had 171 males and 125 females [median age: 72 years (60–78 years)]. Meanwhile, the deceased group consisted of 76 males and 52 females [median age: 74 years (67–81 years)]. Table 1 summarizes the general clinical information of the 424 enrolled patients.

**TABLE 1:** General clinical data and outcomes of Omicron patients.

Variables	Condition on admission					Outcome				
	Mild-to-moderate (n = 76)	Severe-to-critical (n = 348)	Statistics	*p*-Value	Estimated difference, (95%CI)	Survivor (n = 296)	Non-survivor (n = 128)	Statistics	*p*-Value	Estimated difference, (95%CI)
Sex (Male/female)	41/35	206/142	χ^2^ = 0.706	0.401	0.032 (-0.045 to 0.112)^a^	171/125	76/52	χ^2^ = 0.095	0.758	0.014 (-0.079 to 0.104)^a^
Age, y	70.50 (56.25–82.00)	73.00 (64.00–80.00)	Z = 0.918	0.358	−2.000 (-5.000 to 2.000)^b^	71.50 (60.00–78.00)	74.00 (67.00–81.00)	Z = 2.327	0.020	−3.000 (-5.000 to 0.000)^b^
Smoking History	12/64	72/276	χ^2^ = 0.943	0.332	0.045 (-0.059 to 0.124)^a^	62/234	22/106	χ^2^ = 0.795	0.373	0.050 (-0.069 to 0.152)^a^
**Symptoms, n**										
Fever	60/16	275/73	χ^2^ = 0.000	0.988	0.001 (-0.084 to 0.107)^a^	233/63	102/26	χ^2^ = 0.051	0.822	0.012 (-0.105 to 0.116)^a^
Cough	62/14	294/54	χ^2^ = 0.391	0.532	0.032 (-0.064 to 0.156)^a^	264/32	92/36	χ^2^ = 19.894	<0.0001	0.271 (0.138–0.400)^a^
Expectoration	56/20	270/78	χ^2^ = 0.534	0.465	0.032 (-0.053 to 0.136)^a^	249/47	77/51	χ^2^ = 28.880	<0.0001	0.284 (0.170–0.395)^a^
Dyspnea	52/24	313/35	χ^2^ = 24.119	<0.0001	0.264 (0.134–0.404)^a^	246/50	119/9	χ^2^ = 7.253	0.007	0.174 (0.042–0.265)^a^
**Comorbidity, n**										
Hypertension	24/52	176/172	X^2^ = 9.032	0.003	0.112 (0.036–0.186)^a^	128/168	72/56	χ^2^ = 6.066	0.014	0.110 (0.019–0.199)^a^
Diabetes	15/61	116/232	X^2^ = 5.401	0.020	0.094 (0.011–0.164)^a^	83/213	48/80	χ^2^ = 3.745	0.053	0.093 (-0.005 to 0.195)^a^
Cardiovascular Disease	18/58	152/196	X^2^ = 10.382	0.001	0.123 (0.046–0.193)^a^	113/183	57/71	χ^2^ = 1.503	0.220	0.056 (-0.036 to 0.150)^a^
Pulmonary disease	6/70	58/290	X^2^ = 3.745	0.053	0.101 (-0.012 to 0.172)^a^	54/242	10/118	χ^2^ = 7.586	0.006	0.172 (0.045–0.262)^a^
Chronic kidney disease	3/73	22/326	X^2^ = 0.634	0.426	0.063 (-0.144 to 0.161)^a^	14/282	11/117	χ^2^ = 2.405	0.121	0.147 (-0.049 to 0.359)^a^
Malignant tumor	2/74	14/334	X^2^ = 0.333	0.564	0.056 (-0.217 to 0.168)^a^	7/289	9/119	χ^2^ = 5.359	0.021	0.271 (0.010–0.505)^a^
**Lung imaging inflammation site**										
Unilateral/Bilateral	7/69	22/326	X^2^ = 0.817	0.366	0.067 (-0.071 to 0.268)^a^	22/274	7/121	χ^2^ = 0.541	0.462	0.065 (-0.138 to 0.205)^a^
**Laboratory parameters**										
WBC (x10^9^/L)	6.85 (5.05–9.55)	7.60 (5.60–11.38)	Z = 1.889	0.058	−0.90 (-1.80 to 0.00)^b^	6.80 (5.20–9.88)	9.65 (6.60–14.05)	Z = 4.917	<0.0001	−2.30 (-3.20 to −1.40)^b^
LYM (x10^9^/L)	0.90 (0.60–1.40)	0.70 (0.40–1.00)	Z = −3.821	<0.0001	0.20 (0.10–0.30)^b^	0.80 (0.50–1.20)	0.60 (0.40–0.90)	Z = −4.255	<0.0001	0.20 (0.10–0.30)^b^
NE (x10^9^/L)	4.98 (3.57–7.25)	6.39 (4.15–9.89)	Z = 2.754	0.006	−1.18 (-2.07 to −0.34)^b^	5.36 (3.71–8.11)	8.08 (5.62–12.15)	Z = 5.905	<0.0001	−2.61 (-3.46 to −1.77)^b^
EOS (x10^9^/L)	0.02 (0.00–0.12)	0.00 (0.00–0.04)	Z = −4.056	<0.0001	0.01 (0.00–0.02)^b^	0.01 (0.00–0.07)	0.00 (0.00–0.01)	Z = −5.092	<0.0001	0.00 (0.00–0.01)^b^
D-Dimer (ug/mL FE)	0.70 (0.50–1.44)	1.60 (0.89–4.54)	Z = 5.664	<0.0001	−0.72 (-1.06 to 0.45)^b^	1.11 (0.61–2.22)	3.56 (1.50–8.37)	Z = 8.114	<0.0001	−1.65 (-2.48 to −1.12)^b^
PCT (ng/mL)	0.06 (0.04–0.09)	0.14 (0.06–0.67)	Z = 5.810	<0.0001	−0.056 (-0.103 to 0.031)^b^	0.08 (0.05–0.16)	0.48 (0.17–2.93)	Z = 10.059	<0.0001	−0.343 (-0.473 to −0.233)^b^
ALB (g/L)	36.35 (33.70–39.13)	32.35 (29.63–35.40)	Z = −6.787	<0.0001	4.10 (2.90–5.20)^b^	34.45 (31.00–37.30)	31.15 (28.23–33.50)	Z = −6.906	<0.0001	3.40 (2.50–4.40)^b^
LDH (U/L)	215.00 (191.00–269.00)	322.00 (241.25–451.75)	Z = 7.182	<0.0001	−90 (-118 to −64)^b^	250.00 (205.00–329.75)	432.50 (330.75–591.00)	Z = 10.663	<0.0001	−156 (-22.79to −9.42)^b^
CRP (mg/L)	19.63 (2.43–51.44)	50.14 (24.18–60.62)	Z = 5.251	<0.0001	−17.115 (-25.99 to −9.65)^b^	36.74 (9.78–58.13)	56.09 (43.12–61.51)	Z = 5.903	<0.0001	−15.55 (-22.79 to −9.42)^b^
β2-MG (mg/L)	2.79 (1.78–3.45)	3.31 (2.28–5.32)	Z = 3.758	<0.0001	−0.74 (-1.17 to −0.34)^b^	2.84 (2.02–4.00)	4.72 (3.07–10.50)	Z = 7.236	<0.0001	−1.7 (-2.24 to −1.23)^b^

WBC, white blood count; LYM, lymphocyte; NE, neutrophil; EOS, eosinophil; PCT, procalcitonin; ALB, albumin; LDH, lactate dehydrogenase; CRP, C reactive protein; β2-MG, beta-2 microglobulin.

^a^ The rate differences between the two groups were used to express their effect sizes, and the Wilson program to calculate the lower and upper 95% confidence intervals for the difference between the two independent proportions and to correct for this.

^b^ The pseudo-median difference calculated using the Mann-Whitney U test-based Hodges-Lehmann estimation was used as the effect sizes; the confidence interval was calculated using the Mann-Whitney *U* test.

### ROC curve analyses

**FIGURE 2:**
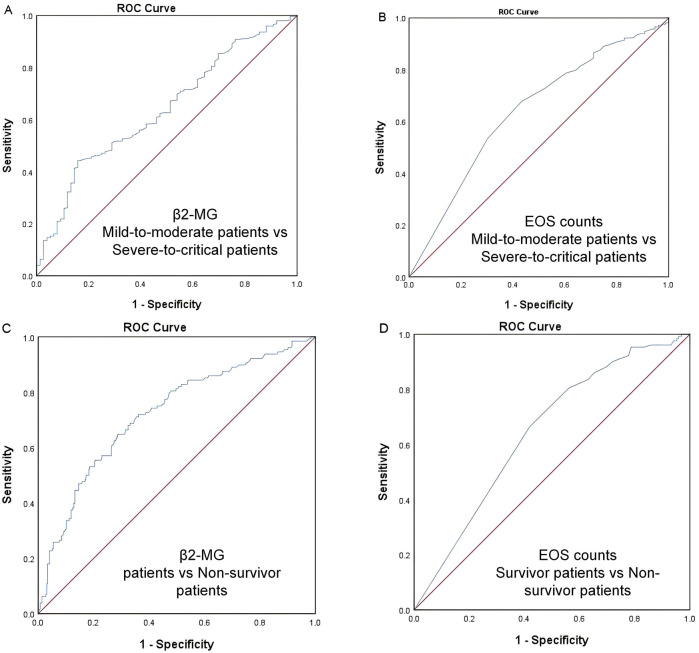
**(A–D)** ROC curve. The ROC analysis provides a cutoff value of 3.655 mg/L for predicting COVID-19 severity **(A)** and 3.625 mg/L for predicting COVID-19 mortality **(B)** using β2-MG. The ROC analysis provides a cutoff value of 0.015 ×10^9^/L for predicting COVID-19 severity **(C)** and 0.005 ×10^9^/L for predicting COVID-19 mortality **(D)** using EOS.

ROC analysis identified a β2-MG cutoff value of 3.655 mg/L for predicting severe and critical disease classification in omicron variant COVID-19 cases, with an area under the receiver operating characteristic (ROC) curve (AUC) of 0.638 (95% CI, 0.572–0.703, *p*<0.0001; Figure 2A).

Similarly, an EOS threshold of 0.015 ×10^9^/L predicted severe and critical disease classification, with an AUC of 0.639 (95% CI, 0.570–0.708, *p*<0.0001; Figure 2B).

For mortality prediction, the ROC analysis set a β2-MG cutoff value of 3.625 mg/L, with an AUC of 0.721 (95% CI 0.667–0.775, *p*<0.0001; Figure 2C).

Additionally, an EOS level of 0.005 ×10^9^/L was determined as a mortality predictor, with an AUC of 0.646 (95% CI, 0.5915–0.700, *p*<0.0001; Figure 2D).

### Serum β2-MG level and EOS count in peripheral blood of patients with omicron

**FIGURE 3:**
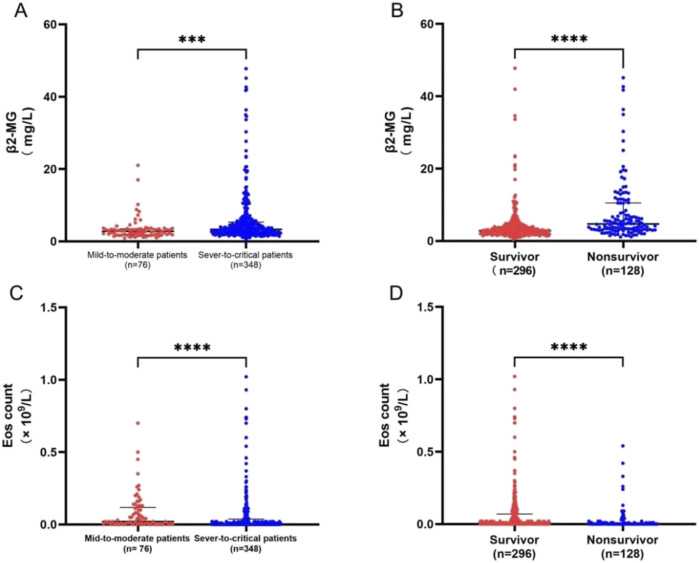
**(A–D) (A)** Contrast serum β2-MG levels between 76 mild-to-moderate and 348 severe-to-critical patients. **(B)** Contrast serum β2-MG levels between 296 survivors and 128 deceased patients. **(C)** Contrast peripheral blood EOS counts between 76 mild-to-moderate and 348 severe-to-critical patients. **(D)** Contrast peripheral blood EOS counts between 296 survivors and 128 deceased patients. ***p < .001; ***p < .0001.

In patients diagnosed with the omicron variant, our analysis revealed that serum β2-MG levels in the peripheral blood were notably higher in critically ill patients than in those with mild-to-moderate symptoms (*P*<0.05). Furthermore, deceased patients displayed elevated β2-MG levels compared to survivors (*P*<0.0001) (Figures 3A, B).

Similarly, EOS levels in the peripheral blood were significantly lower in critically ill patients than in those with mild-to-moderate symptoms. Deceased patients displayed a lower reduction in β2-MG levels than survivors (*P*<0.0001) (Figures 3C, D).

### The relationship between serum β2-MG level and EOS with other factors in clinics and laboratory

In this study, COVID-19 patients were grouped according to the severity of their symptoms, and then further divided into two groups according to their β2-MG levels: those with β2-MG < 3.655 comprised the low β2-MG group, while those with β2-MG ≥3.655 formed the high β2-MG group. The pseudo-median between-group difference in mortality rates between the two groups was 0.357 (95% CI, 0.251 to 0.452, *P* < 0.0001), calculated with the use of the Mann-Whitney *U* test and the Hodges-Lehmann estimate of confidence intervals for pseudo-medians. The pseudo-median between-group difference in significant cough was 0.182 (95% CI, 0.047 to 0.311, *p* = 0.005), in expectoration was 0.128 (95% CI, 0.0123 to 0.243, *p* = 0.0023), in comorbid hypertension was 0.158 (95% CI, 0.062 to 0.251, *p* = 0.001), in underlying pulmonary disease was 0.123 (95% CI, −0.008 to 0.245, *p* = 0.05), in chronic kidney disease was 0.519 (95% CI: (0.310–0.619, *p* < 0.0001), in D-Dimer was −0.75 (95% CI, −1.08 to 0.48, *p* < 0.0001), in PCT was −0.276 (95% CI, −0.431 to −0.184, *p* < 0.0001), in LDH was −69 (95% CI, −94 to −46, *p* < 0.0001), in CRP was −10.450 (95% CI, −15.94 to −5.95, *p* < 0.0001), in lymphocytes was 2.00 (95% CI, 0.10 to 0.20, *p* < 0.0001) and in ALB was 2.9 (95% CI, 1.9 to 3.9, *p* < 0.0001). No other significant differences were observed between the groups (Table 2).

**TABLE 2:** Data categorizing patients based on COVID-19 severity according to β2-MG and EOS levels (The established cut-off value was 3.655 mg/L for β2-MG and 0.015×10^9^/L for EOS).

Variables	Low β2-MG group (n = 269)	High β2-MG group (n = 155)	Statistics	*p*-Value	Estimated difference, (95%CI)	Low EOS group (n = 258)	High EOS group (n = 166)	Statistics	*p*-Value	Estimated difference, (95%CI)
Sex (Male/female)	145/113	102/64	χ^2^ = 1.142	0.285	0.051 (-0.046 to 0.146)^a^	162/107	85/70	χ^2^ = 1.172	0.279	0.051 (-0.044 to 0.148)^a^
Age, y	71.00 (61.00–78.00)	74.00 (64.75–82.00)	Z = 2.699	0.007	−3.00 (-5.00 to −1.00)^b^	73.00 (64.00–80.00)	72.00 (60.00–78; 00)	Z = −1.026	0.305	1.00 (-1.00 to 4.00)^b^
Smoking History	49/209	35/131	χ^2^ = 0.278	0.598	0.031 (-0.087 to 0.155)^a^	50/219	34/121	χ^2^ = 0.694	0.405	0.049 (-0.068 to 0.172)^a^
Survivor/non-survivor, n	46/212	84/82	χ^2^ = 47.763	<0.0001	0.357 (0.251–0.452)^a^	166/103	130/25	χ^2^ = 22.915	<0.0001	0.244 (0.145–0.330)^a^
Fever	205/53	130/36	χ^2^ = 0.080	0.778	0.016 (-0.099 to 0.138)^a^	215/54	120/35	χ^2^ = 0.372	0.542	0.035 (-0.079 to 0.156)^a^
Cough	227/31	129/37	χ^2^ = 7.917	0.005	0.182 (0.047–0.311)^a^	224/45	132/23	χ^2^ = 0.261	0.610	0.033 (-0.103 to 0.152)^a^
Expectoration	208/50	118/48	χ^2^ = 5.169	0.023	0.128 (0.0123–0.243)^a^	200/69	126/29	χ^2^ = 2.666	0.103	0.091 (-0.024 to 0.193)^a^
Dyspnea	219/39	146/20	χ^2^ = 0.794	0.373	0.061 (-0.084 to 0.187)^a^	246/23	119/36	χ^2^ = 17.681	<0.0001	0.284 (0.139–0.415)^a^
Hypertension	105/153	95/71	χ^2^ = 11.077	0.001	0.158 (0.062–0.251)^a^	141/128	59/96	χ^2^ = 8.128	0.004	0.134 (0.039–0.225)^a^
Diabetes	75/183	56/110	χ^2^ = 1.030	0.310	0.052 (-0.051 to 0.157)^a^	93/176	38/117	χ^2^ = 4.658	0.031	0.109 (0.006–0.204)^a^
Cardiovascular disease	98/160	72/94	χ^2^ = 1.221	0.269	0.054 (-0.044 to 0.151)^a^	115/154	55/100	χ^2^ = 2.162	0.141	0.070 (-0.027 to 0.163)^a^
Pulmonary disease	46/212	18/148	χ^2^ = 3.847	0.050	0.123 (-0.008 to 0.245)^a^	39/230	25/130	χ^2^ = 0.204	0.651	0.030 (-0.099 to 0.169)^a^
Chronic kidney disease	3/255	22/144	χ^2^ = 26.610	<0.0001	0.519 (0.310–0.619)^a^	17/252	8/147	χ^2^ = 0.238	0.626	0.048 (-0.172 to 0.219)^a^
Malignant tumor	6/252	10/156	χ^2^ = 3.805	0.051	0.243 (-0.028 to 0.460)^a^	11/258	5/150	χ^2^ = 0.202	0.653	0.055 (-0.222 to 0.253)^a^
Unilateral/Bilateral	18/240	11/155	χ^2^ = 0.019	0.889	0.001 (-0.178 to 0.206)^a^	20/249	9/146	χ^2^ = 0.409	0.522	0.0593 (-0.1455 to 0.2179)^a^
WBC (x10^9^/L)	7.35 (5.70–10.50)	7.75 (5.08–11.40)	Z = 0.373	0.709	−2.00 (-1 to 0.70)^b^	7.50 (5.30–11.40)	7.40 (5.80–10.70)	Z = 0.295	0.768	−0.10 (-0.90 to 0.70)^b^
LYM (x10^9^/L)	0.80 (0.50–1.23)	0.60 (0.40–0.90)	Z = −3.608	<0.0001	2.00 (0.10–0.20)^b^	0.60 (0.40–0.80)	1.00 (0.70–1.50)	Z = 8.687	<0.0001	−0.40 (-0.50 to −0.30)^b^
NE (x10^9^/L)	5.80 (4.06–9.14)	6.83 (4.06–9.89)	Z = 1.443	0.149	−0.57 (-1.33 to 0.21)^b^	6.40 (4.16–10.16)	5.57 (3.85–9.28)	Z = −1.807	0.071	0.66 (-0.06 to 1.40)^b^
EOS (x10^9^/L)	0.01 (0.00–0.07)	0.00 (0.00–0.02)	Z = −2.715	0.007	0.000 (0.00–0.00)^b^	0.00 (0.00–0.00)	0.08 (0.04–0.16)	Z = 18.318	<0.0001	−0.08 (-0.09 to −0.07)^b^
D-Dimer (ug/mL FE)	1.12 (0.59–2.58)	2.16 (1.17–5.31)	Z = 5.471	<0.0001	−0.75 (-1.08 to 0.48)^b^	1.60 (0.89–4.68)	1.17 (0.61–2.52)	Z = −3.461	0.001	0.41 (0.17–0.70)^b^
PCT (ng/mL)	0.07 (0.05–0.13)	0.44 (0.13–2.88)	Z = 10.644	<0.0001	−0.276 (-0.431 to −0.184)^b^	0.15 (0.06–0.67)	0.08 (0.05–0.19)	Z = −4.015	<0.0001	0.034 (0.016–0.063)^b^
ALB (g/L)	34.20 (31.10–37.13)	31.75 (28.05–34.80)	Z = −5.912	<0.0001	2.9 (1.9–3.9)^b^	32.60 (30.00–35.50)	34.60 (30.60–37.70)	Z = 3.316	0.001	−1.70 (-2.70 to −0.70)^b^
LDH (U/L)	267.00 (206.75–363.25)	340.50 (262.25–502.25)	Z = 5.570	<0.0001	−69 (-94 to −46)^b^	332.00 (252.50–494.50)	233.00 (195.00–316.00)	Z = −7.677	<0.0001	86.00 (64.00–110.00)^b^
CRP (mg/L)	36.83 (8.08–57.80)	53.41 (36.39–60.97)	Z = 4.933	<0.0001	−10.450 (-15.94 to −5.95)^b^	50.30 (24.34–60.65)	36.68 (7.01–57.62)	Z = −3.808	<0.0001	8.03 (3.45–13.47)^b^
β2-MG (mg/L)	2.37 (1.82–3.05)	5.97 (4.49–11.04)	Z = 17.387	<0.0001	−3.6 (-4.13 to −3.18)^b^	3.33 (2.31–5.32)	2.87 (2.03–4.38)	Z = −2.816	0.005	0.47 (0.14–0.81)^b^

WBC, white blood count; LYM, lymphocyte; NE, neutrophil; EOS, eosinophil; PCT, procalcitonin; ALB, albumin; LDH, lactate dehydrogenase; CRP, C reactive protein; β2-MG, beta-2 microglobulin.

^a^ The rate differences between the two groups were used to express their effect sizes, and the Wilson program to calculate the lower and upper 95% confidence intervals for the difference between the two independent proportions and to correct for this.

^b^ The pseudo-median difference calculated using the Mann-Whitney U test-based Hodges-Lehmann estimation was used as the effect sizes; the confidence interval was calculated using the Mann-Whitney *U* test.

Similarly, patients were categorized based on EOS levels: EOS < 0.015 × 10^9^/L constituted the low EOS group, and EOS ≥ 0.015 × 10^9^/L represented the high EOS group. The pseudo-median between-group difference in mortality rates between the two EOS groups was 0.244 (95% CI, 0.145 to 0.330, *p* < 0.0001), in significant dyspnea symptoms was 0.244 (95% CI, 0.145 to 0.330, *p* < 0.0001), in more comorbid hypertension was 0.134 (95% CI, 0.039 to 0.225, *p* = 0.004), in diabetes was 0.109 (95% CI, 0.006 to 0.204, *p* = 0.031), in D-Dimer was 0.41 (95% CI, 0.17 to 0.70, *p* < 0.000), and in PCT was 0.034 (95% CI, 0.016 to 0.063, *p* < 0.0001), in LDH was 86.00 (95% CI, 64.00 to 110.00, *p* < 0.0001), in CRP was 8.03 (95% CI, 3.45 to 13.47, *p* < 0.0001), in β2-MG was 0.47 (95% CI, 0.14 to 0.81, *p* = 0.005), in Lymphocytes was −0.40 (95% CI, −0.50 to −0.30, *p* < 0.0001) and in ALB was −1.70 (95% CI,-2.70 to 0.70, *p* < 0.0001). No significant differences were observed in other factors between the two groups (Table 2).

Furthermore, in this study, we categorized COVID-19 patients based on their outcomes. Patients were divided into two groups based on β2-MG levels: those with β2-MG <3.625 mg/L formed the low β2-MG group, while those with β2-MG ≥ 3.625 mg/L constituted the high β2-MG group. The findings revealed that compared to the low β2-MG group, the high β2-MG group was characterized by older age (*p* = 0.004), increased mortality rates (*p* < 0.0001), more pronounced coughing symptoms (*p* = 0.017), and a higher likelihood of comorbidities such as hypertension (*p* < 0.0001), underlying lung disease (*p* = 0.041), chronic kidney disease (*p* < 0.0001). Additionally, the high β2-MG group exhibited significantly lower levels of lymphocytes (*p* < 0.0001), EOS (*p* = 0.004), ALB (*p* < 0.0001), along with elevated levels of D-Dimer (*p* < 0.0001), PCT (*p* < 0.0001), LDH (*p* < 0.0001), and CRP (*p* < 0.0001). No significant differences were observed in other factors between the two groups (Table 3).

**TABLE 3:** Data on β2-MG and EOS levels in COVID-19 patients stratified by outcomes (Using a β2-MG threshold of 3.625 mg/L and an EOS threshold of 0.005×10^9^/L).

Variables	Condition on admission				Outcomes			
	Low β2-MG group (n = 256)	High β2-MG group (n = 168)	Statistics	*p*-Value	Low EOS group (n = 209)	High EOS group (n = 215)	Statistics	*p*-Value
Sex (Male/female)	144/112	103/65	χ^2^ = 1.068	0.301	128/81	119/96	χ^2^ = 1.515	0.218
Age, y	71.00 (61.00–78.00)	74.00 (65.00–82.00)	Z = 2.904	0.004	73.00 (66.00.∼80.00)	71.00 (60.00–79.00)	Z = −1.998	0.046
Smoking History	49/207	35/133	χ^2^ = 0.183	0.669	42/167	42/173	χ^2^ = 0.021	0.885
Survivor/non-survivor, n	45/211	83/85	χ^2^ = 48.752	<0.0001	124/85	172/43	χ^2^ = 21.484	<0.0001
Fever	203/53	132/36	χ^2^ = 0.032	0.858	164/45	171/44	χ^2^ = 0.073	0.788
Cough	225/31	131/37	χ^2^ = 7.404	0.007	176/33	180/35	χ^2^ = 0.019	0.891
Expectoration	207/49	119/49	χ^2^ = 5.738	0.017	154/55	172/43	χ^2^ = 2.379	0.123
Dyspnea	217/39	148/20	χ^2^ = 0.939	0.333	192/17	173/42	χ^2^ = 11.500	0.001
Hypertension	103/153	97/71	χ^2^ = 12.471	<0.0001	113/96	87/128	χ^2^ = 7.868	0.005
Diabetes	73/183	58/110	χ^2^ = 1.715	0.190	76/133	55/160	χ^2^ = 5.771	0.016
Cardiovascular disease	97/159	73/95	χ^2^ = 1.306	0.253	97/112	73/142	χ^2^ = 6.848	0.009
Pulmonary disease	46/210	18/150	χ^2^ = 4.165	0.041	29/180	35/180	χ^2^ = 0.478	0.489
Chronic kidney disease	3/253	22/146	χ^2^ = 25.990	<0.0001	12/197	13/202	χ^2^ = 0.018	0.894
Malignant tumor	6/250	10/158	χ^2^ = 3.638	0.056	8/201	8/207	χ^2^ = 0.003	0.954
Unilateral/Bilateral	18/238	11/157	χ^2^ = 0.037	0.847	17/192	12/203	χ^2^ = 1.084	0.298
WBC (x10^9^/L)	7.35 (5.70–10.50)	7.75 (5.13–11.38)	Z = 0.402	0.688	7.60 (5.35–11.15)	7.40 (5.60–10.90)	Z = 0.019	0.985
LYM (x10^9^/L)	0.80 (0.50–1.28)	0.60 (0.40–0.90)	Z = −3.602	<0.0001	0.60 (0.40–0.80)	0.90 (0.60–1.40)	Z = 7.615	<0.0001
NE (x10^9^/L)	5.72 (4.05–9.17)	6.83 (4.08–9.86)	Z = 1.484	0.138	6.55 (4.21–10.02)	5.62 (3.87–9.32)	Z = −1.491	0.136
EOS (x10^9^/L)	0.01 (0.00–0.07)	0.00 (0.00–0.02)	Z = −2.896	0.004	0.00 (0.00–0.00)	0.04 (0.01–0.13)	Z = 19.017	<0.0001
D-Dimer (ug/mL FE)	1.12 (0.59–2.63)	2.09 (1.16–5.30)	Z = 5.383	<0.0001	1.59 (0.90–4.45)	1.25 (0.64–3.24)	Z = −2.345	0.019
PCT (ng/mL)	0.07 (0.05–0.13)	0.41 (0.13–2.80)	Z = 10.529	<0.0001	0.17 (0.06–0.88)	0.09 (0.05–0.26)	Z = −3.810	<0.0001
ALB (g/L)	34.20 (31.13–37.18)	31.75 (27.95–34.80)	Z = −5.939	<0.0001	32.60 (30.00–35.40)	34.10 (30.30–37.00)	Z = 2.375	0.018
LDH (U/L)	265.00 (206.25–363.00)	340.50 (263.50–498.50)	Z = 5.626	<0.0001	337.00 (250.00–494.50)	260.00 (205.00–347.00)	Z = −5.672	<0.0001
CRP (mg/L)	36.83 (8.20–57.72)	53.41 (35.92–61.10)	Z = 4.880	<0.0001	51.04 (27.49–60.84)	40.69 (8.58–57.93)	Z = −3.725	<0.0001
β2-MG (mg/L)	2.37 (1.82–3.03)	5.88 (4.45–10.98)	Z = 17.424	<0.0001	3.31 (2.24–5.53)	3.05 (2.18–4.49)	Z = −1.875	0.061

WBC, white blood count; LYM, lymphocyte; NE, neutrophil; EOS, eosinophil; PCT, procalcitonin; ALB, albumin; LDH, lactate dehydrogenase; CRP, C reactive protein; β2-MG, beta-2 microglobulin.

Similarly, patients were also segregated based on EOS levels: the low EOS group had levels <0.005 ×10^9^/L, while the high EOS groups had levels ≥0.005×10^9^/L. The results indicated that the low EOS group was older (*p* = 0.046), had a higher mortality rate (*p* < 0.0001), and more pronounced dyspnea (*p* = 0.001). Additionally, this group exhibited increased comorbidities such as hypertension (*p* = 0.005), diabetes mellitus (*p* = 0.016), coronary artery disease (*p* = 0.009), D-Dimer (*p* = 0.019), PCT (*p* < 0.0001), LDH (*p* < 0.0001), and CRP (*p* < 0.0001) levels, along with reduced levels of lymphocytes (*p* < 0.0001) and ALB (*p* = 0.018). No significant differences were observed in other factors between the two groups (Table 3).

**FIGURE 4:**
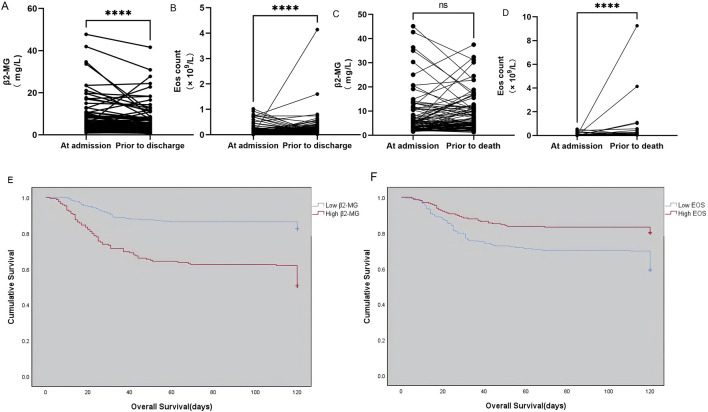
**(A–F)** Dynamics of serum β2-MG **(A)** and peripheral blood EOS count **(B)** in omicron patients whose viral nucleic acid test turned negative before discharge. The dynamic changes of serum β2-MG **(C)** and peripheral blood EOS count **(C)** in omicron patients before death. Overall survival based on serum β2-MG **(E)** and peripheral blood EOS count **(F)** in omicron patients.

In this study, we monitored patients diagnosed with omicron mutation, focusing on the outcome of nucleic acid turned negative upon discharge. We observed the changes in β2-MG and eosinophil levels before discharge and noted that a majority of patients exhibited significant improvements in β2-MG levels (Figure 4A), while their eosinophil levels recovered (Figure 4B). For patients with death as the outcome, we monitored their β2-MG and eosinophil levels leading up to their demise. Our findings revealed that the majority exhibited stable β2-MG levels (Figure 4C) while showing a decline in eosinophil levels (Figure 4D) before their death.

### Survival rate analysis

The study categorized patients diagnosed with omicron variants who faced fatal outcomes. The mean OS was significantly shorter in the group with high β2-MG levels (256) compared to those with low β2-MG levels (168) (107.234 days vs 84.107 days, χ^2^ = 51.526, *p* < 0.0001; Figure 4E). The mean OS was significantly shorter in the low EOS level group (209) compared with the high EOS level group (215) (91.751 days vs 104.214 days, χ^2^ = 20.237, *p* < 0.0001; Figure 4F).

### Poor prognosis linked to elevated β2-MG levels and reduced EOS levels

Before conducting the COX regression analysis, we conducted tests on the proportional hazards assumption for the included variables. For the variables that did not satisfy the assumption of proportional hazards, we carried out Post hoc subgroup analysis and adopted the method of subgroup analysis to further explore their influences. Univariate analysis indicated that OS correlated with factors such as older age (*p* = 0.021), symptoms of cough (*p* < 0.0001), sputum (*p* < 0.0001), and dyspnea (*p* = 0.012). Additionally, comorbidities including hypertension (*p* = 0.015), diabetes mellitus (*p* = 0.045), underlying lung disease (*p* = 0.013), and malignancy (*p* = 0.029) were associated with OS. Elevated levels of WBC (*p* < 0.0001), NE (*p* < 0.0001), D-Dimer (*p* = 0.0001), PCT (*p* < 0.0001), CRP (*p* = 0.0001), LDH (*p* < 0.0001), and higher β2-MG (*p* < 0.0001) were also linked with a poorer prognosis. Conversely, reduced levels of EOS (*p* < 0.0001), lymphocytes (*p* = 0.007), and ALB (*p* = 0.004) were negatively associated with OS (Table 4).

**TABLE 4:** Univariate and multivariate analyses of prognostic parameters for overall survival in omicron patients.

Variables	Univariate analysis for OS			Multivariate analysis for OS		
	HR	95% CI	*p*-value	HR	95% CI	*p*-value
Sex (Male)	1.045	0.735–1.487	0.806			
Age≥60 (years)	1.883	1.099–3.227	0.021*	1.646	0.938–2.887	0.082
Smoking History	0.779	0.492–1.232	0.285			
Fever	1.016	0.661–1.564	0.941			
Cough	0.398	0.271–0.585	<0.0001*	0.781	0.414–1.473	0.445
Expectoration	0.374	0.263–0.533	<0.0001*	0.665	0.372–1.187	0.168
Dyspnea	2.382	1.210–4.692	0.012*	1.382	0.658–2.902	0.393
Hypertension	1.541	1.087–2.185	0.015*	1.695	0.779–1.695	0.484
Diabetes	1.442	1.008–2.062	0.045*	1.127	0.770–1.649	0.540
Cardiovascular disease	1.284	0.906–1.819	0.161			
Pulmonary disease	2.274	1.192–4.337	0.013*	0.886	0.452–1.736	0.724
Chronic kidney disease	1.711	0.922–3.176	0.089*	1.202	0.62–2.329	0.586
Malignant tumor	2.126	1.079–4.190	0.029*	1.673	0.796–3.521	0.175
WBC≥9.5×10^9^/L	2.254	1.593–3.189	<0.0001*	0.994	0.594–1.663	0.982
Lym <1.1×10^9^/L	1.873	1.183–2.964	0.007*	1.153	0.706–1.884	0.570
NE ≥ 6.3×10^9^/L	2.681	1.845–3.898	<0.0001*	1.358	0.800–2.304	0.257
EOS <0.005×10^9^/L	2.248	1.557–3.244	<0.0001*	1.292	0.867–1.928	0.209
D-Dimer≥0.5ug/ML FE	6.733	2.142–21.161	0.001*	1.920	0.582–6.334	0.284
PCT≥0.5 ng/mL	3.569	2.522–5.051	<0.0001*	1.591	1.025–2.469	0.038*
CRP≥5 mg/L	4.877	1.994–11.928	0.001*	1.413	0.545–3.663	0.477
ALB <40 g/L	8.009	1.981–32.377	0.004*	1.819	0.430–7.688	0.416
LDH≥250 U/L	14.537	6.403–33.002	<0.0001*	7.801	3.343–18.201	<0.0001*
β2-MG≥3.625 mg/L	3.441	2.393–4.948	<0.0001*	1.620	1.050–2.499	0.029*

WBC, white blood count; LYM, lymphocyte; NE, neutrophil; EOS, eosinophil; PCT, procalcitonin; ALB, albumin; LDH, lactate dehydrogenase; CRP, C reactive protein; β2-MG, beta-2 microglobulin.

**FIGURE 5:**
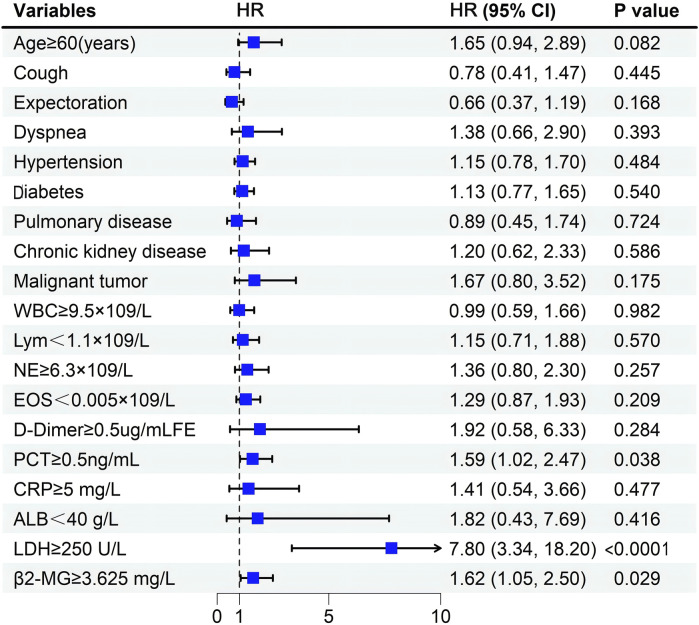
Forest plot for multifactor Cox regression analysis.

Multivariate analysis revealed that elevated PCT (*p* = 0.038), LDH (*p* < 0.0001), and β2-MG (*p* = 0.029) levels independently correlated with a poorer OS (Table 4; Figure 5).

**FIGURE 6:**
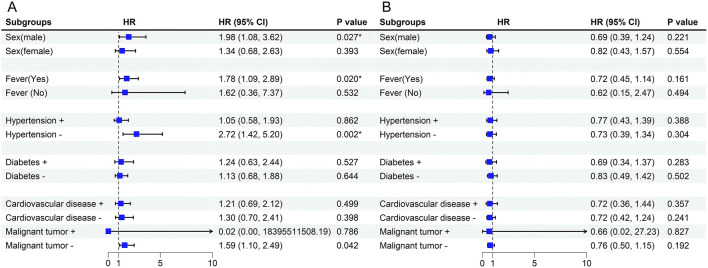
**(A)** Forest plot for Stratification Analyses of the association between serum β2-MG and mortality in Omicron patients. **(B)** Forest plot for Stratification Analyses of the association between EOS and mortality in Omicron patients. Among the patients with malignant tumors, n = 16 (3.77%), the sample size was too small, resulting in a wider 95%CI, and the result was uncertain.

For variables that did not conform to the proportional risk hypothesis, we further analyzed risk stratification values for β2-MG and EOS levels for primary endpoints in multiple subgroups, including sex, fever, comorbidized hypertension, diabetes, Cardiovascular disease, and Malignant tumor (Figure 6). The results showed that elevated serum β2-MG levels were significantly associated with higher risk of hospital mortality in male patients 1.977 (95%CI, 1.079–3.621,*p* = 0.027), patients with fever symptoms 1.778 (95%CI, 1.093–2.894,*p* = 0.020), and patients without hypertension 2.721 (95%CI, 1.424–5.200,*p* = 0.002), or without malignancy 1.590 (95%CI, 1.1.107–2.487,*p* = 0.042).

## Discussion

The research results show that serum β2-MG levels are elevated in critically ill Omicron patients, while peripheral blood EOS counts are reduced. We found that the β2-MG levels in patients with fatal outcomes were higher than those in surviving patients at admission, while the EOS counts were the opposite. Additionally, compared to when nucleic acid tests were positive, patients showed decreased β2-MG levels and increased EOS counts after the tests turned negative. Notably, the serum β2-MG levels in patients who died did not show significant changes before death compared to admission, while EOS levels decreased before death. This suggests that β2-MG may be involved in the pathogenesis of COVID-19 in patients with the Omicron variant. We noted that during the ROC curve analysis, the AUC values of 0.638 and 0.646 indicate moderate predictive performance; however, we believe that even a moderate level of predictive ability is clinically significant, particularly when combined with other clinical parameters. We emphasize that β2-MG and eosinophil levels can serve as complementary biomarkers, enhancing clinical decision-making within a broader diagnostic framework. Furthermore, we noted that in a recent 65-day short-term study on the Omicron variant of COVID-19, the reported AUC value for β2-MG was 0.638 (Gong et al., 2023), confirming its utility in diagnosing the severity of Omicron infections, which is consistent with our findings. We will discuss the implications of these findings for clinical practice, including considerations for patient stratification and the potential of these biomarkers to guide treatment decisions.

The results of Cox regression analysis showed that elevated levels of β2-MG, PCT, and LDH were important independent prognostic factors in omicron patients. In the subgroup analysis, we found that elevated serum β2-MG levels were associated with an increased risk of mortality in specific subgroups, such as male patients, those with fever symptoms, and individuals without comorbid hypertension or malignancies. This finding provides a more nuanced perspective on our results and supports the potential role of β2-MG as a prognostic indicator. Although the *p*-value for EOS count was less than 0.05 in the univariate Cox regression analysis, it was greater than 0.05 in the multivariate regression analysis and *post hoc* subgroup analysis, suggesting that its association with mortality in COVID-19 patients infected with the Omicron variant may not be robust. Given these conflicting results, we emphasize the need for further research to deepen our understanding of EOS dynamics in COVID-19 and to validate our findings in larger, more diverse populations.

Present in nearly all nucleated cells, β2-MG is a surface protein that plays a key role in the immune system and glomerular regulation of homeostasis. Research involving β2-MG frequently serves as a tool to assess both glomerular and tubular function in patients diagnosed with kidney-related diseases (Sivanathan et al., 2022). In addition to reacting to kidney-related disease, β2-MG in the blood is used to evaluate kidney function and tumor growth in patients with diffuse large B-cell lymphoma (Kanemasa et al., 2017). Moreover, research has highlighted its sensitivity as a diagnostic marker for a range of tumors, as well as inflammatory and infectious diseases (Bethea and Forman, 1990). β2-MG regulates immune activities in the body, such as immune recognition and immunoglobulin transport (Vianello et al., 2015; Wang et al., 2020). Viral infections such as EBV, influenza, and CMV can lead to elevated β2-MG expression (Cooper et al., 1984). Furthermore, studies have shown that β2-MG can predict the prognosis of patients with various malignant tumors, such as colorectal cancer and Burkitt’s lymphoma (Kim et al., 2021; Na et al., 2021). These results are consistent with our findings. Thus, β2-MG can participate in the inflammatory response and immune activity in the organism, especially reflecting the pathogenesis in patients with hematologic and immune system diseases. Moreover, elevated β2-MG levels typically suggest that patients have advanced disease and a poor prognosis. Conversely, patients with lower β2-MG levels have a relatively better prognosis. The present study showed that omicron patients with higher β2-MG levels had a higher risk of death and shorter survival. Moreover, β2-MG is easy to measure and has good consistency as an indicator of blood biochemistry and renal function in hospitalized patients. Consequently, we posit that β2-MG levels could aid clinicians in making informed therapeutic decisions and predicting clinical prognosis in routine diagnosis and treatment.

EOS originate from pluripotent hematopoietic stem cells within the bone marrow microenvironment, giving rise to distinct eosinophilic progenitor cells that eventually differentiate into mature EOS (Klion et al., 2020). These cells synthesize numerous toxic granule proteins. Following post-translational modification and sequestration, these proteins uphold cell viability and standard physiological function. In addition to their involvement in skin and respiratory diseases, EOS plays a role in a wide range of diseases, including gastrointestinal diseases, cancer, autoimmune diseases, and blood disorders (O'Sullivan and Bochner, 2018). EOS is significantly increased in parasitic infections and allergic diseases (Rosenberg and Domachowske, 2001), and significantly decreased in patients with acute infectious diseases such as typhoid fever (with major surgery and burns) and sepsis (Hassani et al., 2020). Decreased eosinophilia in the blood has also been associated with viral infections (Klion et al., 2020). Decreased eosinophilia in the peripheral blood has been found in studies related to COVID-19, and EOS is involved in antiviral defense responses in the early stages of the disease. *In vivo* experiments have shown that EOS plays a major protective role after infection of mice with RSV and influenza A viruses (Percopo et al., 2014; Samarasinghe et al., 2017). EOS can be recruited to the lungs along with neutrophils and participate in the body’s antiviral host defense (Rosenberg and Domachowske, 2001). Percopo et al. (2014) used a Th2 cytokine-driven mouse model of asthma inflammation to find that EOS has antiviral effects and promotes the survival of lethal pneumovirus in infected mice. Considering the high risk of infection, BALF samples from mildly ill patients were not collected in this study, and the number of EOS was not counted in sputum from mildly ill patients or in BALF samples from severely ill patients. Researchers have hypothesized that large numbers of peripheral blood neutrophils may be recruited to the lungs in patients during COVID-19 infection, thereby accelerating neutrophil production in the bone marrow. As a result of the change in neutrophil production, EOS production may be reduced. Although EOS is reduced regardless of severity, the severe reduction in EOS counts in critically ill patients may be related to the increased secretion of corticosteroids by the adrenal glands during acute lung injury in response to stress, which in turn inhibits EOS release from the bone marrow (Xie et al., 2021). Additionally, our prior research indicated that a progressive decline in eosinophilia aligns with the deterioration of critical illness in COVID-19 patients and is associated with notably higher mortality rates (Yan et al., 2021).

This study showed that 63.83% of patients with omicron infection had reduced EOS levels and 81.25% of patients who died had low EOS levels on admission. A previous study showed that EOS counts were lower in the peripheral blood of COVID-19 patients compared to other types of pneumonia (Xie et al., 2021). Our study found that EOS counts in patients with omicron were indeed lower than the normal range and varied in different degrees of omicron infection, as well as being able to indicate a poor prognosis for the patient. Another study mentioned that counts did not differ significantly between critically and non-critically ill COVID-19 patients (Zhang et al., 2020), but in our study, we found that the EOS levels of critically ill patients were lower than those of non-critically ill patients, and the admission EOS levels of deceased patients were lower than those of surviving patients. This may be related to the fact that the type of virus in its study was SARS-CoV-2 and its inclusion of only 140 samples may be a limitation.

As a result, the above prediction model can be used to determine the poor prognosis of omicron critically ill patients at the time of hospitalization, especially interhospital mortality, and to give more aggressive diagnostic and therapeutic means, such as mechanical ventilation, etc., and to give timely interventions with drugs such as Nirmatrelvir/ritonavir, etc., to reduce the mortality rate, if they are eligible for medication use. Our previous research also indicated that early corticosteroid therapy can reduce mortality rates in severely ill COVID-19 patients (Li et al., 2021).

This study has some limitations. Firstly, the retrospective nature of this research, based on clinical data, introduces potential selection biases and confounding variables. Moreover, the data collection was confined to a single center, suggesting the need for broader, multicenter studies in subsequent research endeavors. Secondly, the study did not undertake an exhaustive investigative analysis to delineate the intricate association between β2-MG and EOS levels with the severity and prognosis of Omicron variant COVID-19. And, while elevated levels of serum β2-MG and EOS offer insights into prognosis, they do not serve as standalone diagnostic markers for COVID-19. Finally, we performed *post hoc* subgroup analyses of COX regression analyses to help better understand the effects of variables in specific populations, but there may be some selection bias and false-positive results, so future studies can further confirm these findings from prospective cohort studies or randomized controlled trials.

## Conclusion

Our study revealed that heightened serum β2-MG and diminished EOS levels were observed in individual Omicron patients. Furthermore, these elevated β2-MG and decreased eosinophil levels correlated with increased disease severity and unfavorable prognoses among Omicron patients. Utilizing serum β2-MG and eosinophil count as prognostic markers could enhance the evaluation of Omicron patient outcomes. These insights not only shed light on the pathophysiology of the Omicron variant but also pave the way for potential therapeutic interventions. However, the prognostic potential of β2-MG and eosinophils should be further explored through pivotal prospective trials, given that our current findings stem from a retrospective analysis primarily designed to formulate hypotheses.

## New and noteworthy

This study found that elevated β2-MG and decreased EOS were associated with severe and adverse outcomes in Omicron patients. The prognostic application of β2-MG and eosinophil counts enhanced the outcome assessment.

## Data Availability

The original data presented in the study are included in the article. Further inquiries can be directed to the corresponding author.
